# AI in Qualitative Health Research Appraisal: Comparative Study

**DOI:** 10.2196/72815

**Published:** 2025-07-08

**Authors:** August Landerholm

**Affiliations:** 1 Physiotherapy Department, Healthscience Faculty, Mälardalen University, Avdelningen för Fysioterapi Akademin för Hälsa, Vård Och Välfärd Mälardalens Universitet, Västerås, 721 21, Sweden, 46 702129863

**Keywords:** artificial intelligence, qualitative research appraisal, systematic reviews, interrater agreement, CASP checklist, Critical Appraisal Skills Programme, JBI checklist, Joanna Briggs Institute, ETQS, Evaluative Tools for Qualitative Studies, large language models, affirmation bias, human-AI collaboration

## Abstract

**Background:**

Qualitative research appraisal is crucial for ensuring credible findings but faces challenges due to human variability. Artificial intelligence (AI) models have the potential to enhance the efficiency and consistency of qualitative research assessments.

**Objective:**

This study aims to evaluate the performance of 5 AI models (GPT-3.5, Claude 3.5, Sonar Huge, GPT-4, and Claude 3 Opus) in assessing the quality of qualitative research using 3 standardized tools: Critical Appraisal Skills Programme (CASP), Joanna Briggs Institute (JBI) checklist, and Evaluative Tools for Qualitative Studies (ETQS).

**Methods:**

AI-generated assessments of 3 peer-reviewed qualitative papers in health and physical activity–related research were analyzed. The study examined systematic affirmation bias, interrater reliability, and tool-dependent disagreements across the AI models. Sensitivity analysis was conducted to evaluate the impact of excluding specific models on agreement levels.

**Results:**

Results revealed a systematic affirmation bias across all AI models, with “Yes” rates ranging from 75.9% (145/191; Claude 3 Opus) to 85.4% (164/192; Claude 3.5). GPT-4 diverged significantly, showing lower agreement (“Yes”: 115/192, 59.9%) and higher uncertainty (“Cannot tell”: 69/192, 35.9%). Proprietary models (GPT-3.5 and Claude 3.5) demonstrated near-perfect alignment (Cramer *V*=0.891; *P*<.001), while open-source models showed greater variability. Interrater reliability varied by assessment tool, with CASP achieving the highest baseline consensus (Krippendorff α=0.653), followed by JBI (α=0.477), and ETQS scoring lowest (α=0.376). Sensitivity analysis revealed that excluding GPT-4 increased CASP agreement by 20% (α=0.784), while removing Sonar Huge improved JBI agreement by 18% (α=0.561). ETQS showed marginal improvements when excluding GPT-4 or Claude 3 Opus (+9%, α=0.409). Tool-dependent disagreements were evident, particularly in ETQS criteria, highlighting AI’s current limitations in contextual interpretation.

**Conclusions:**

The findings demonstrate that AI models exhibit both promise and limitations as evaluators of qualitative research quality. While they enhance efficiency, AI models struggle with reaching consensus in areas requiring nuanced interpretation, particularly for contextual criteria. The study underscores the importance of hybrid frameworks that integrate AI scalability with human oversight, especially for contextual judgment. Future research should prioritize developing AI training protocols that emphasize qualitative epistemology, benchmarking AI performance against expert panels to validate accuracy thresholds, and establishing ethical guidelines for disclosing AI’s role in systematic reviews. As qualitative methodologies evolve alongside AI capabilities, the path forward lies in collaborative human-AI workflows that leverage AI’s efficiency while preserving human expertise for interpretive tasks.

## Introduction

### Importance of Quality Assessment in Qualitative Research

Systematic quality assessment is foundational for establishing the credibility, dependability, and transferability of qualitative research findings [1]. Rigorous appraisal enables readers to evaluate the trustworthiness of study conclusions and their applicability to real-world contexts. Unlike quantitative methodologies, qualitative research prioritizes contextual richness and interpretive depth, necessitating frameworks that account for methodological diversity across paradigms (eg, phenomenology and ethnography).

Cross-study comparison is crucial for building a robust evidence base in any given field; yet, qualitative studies have seen little attention in systematic reviews. Facilitating and synthesizing research in qualitative methodologies is challenging because these approaches are based on diverse philosophical foundations, such as phenomenology, ethnography, and grounded theory, as well as a wide variety of analytical methods [2,3]. The chosen analysis method of each research group may have varying degrees of subjective data interpretation, leading to diverse findings and conclusions [4]. Further complicating is the contextual nature of each study, where the findings may be highly dependent on its unique context [3]. The richness of data in qualitative datasets also encourages attempts at cohesive summaries, which can be challenging without losing important details or context [1]. Qualitative studies typically use smaller sample sizes than their quantitative methodological counterparts and use purposively selected samples [5]. Depending on the degree of rigor in the selection criteria, it becomes difficult to determine transferability or comparability [6]. Facilitating comparison and research synthesis becomes easier through standardized quality assessment criteria.

### Systematic Assessment for Qualitative Research

Various assessment tools exist for systematically addressing qualitative study quality and have all been developed to address the unique challenges of evaluating qualitative research [7]. If a research group wishes to systematically address a field that has been given qualitative attention, their choice of assessment tool will provide different weight to studies in the final synthesis, potentially affecting the review’s conclusions. There are also potential limitations of an overly systematic approach to addressing qualitative study quality, as overly rigid quality criteria may not capture the diversity of qualitative research approaches more broadly [2].

There are methodological issues with systematic quality assessment that endanger the potential credibility of a systematic review. Quality assessment of qualitative research is often time-consuming and labor-intensive, limiting the number of studies that systematic reviews tend to include. Human reviewers may have varying interpretations of quality criteria, leading to inconsistencies [8]. Different research groups might dissolve this in different ways, with bias present [9]. Humans’ ability to recognize patterns is ever so present in systematic reviews, as a reviewer can detect recurring themes or quality indicators across supposedly independent quality assessments. These aspects may be augmented using another, nonhuman, reviewer [10].

In the context of health research, the systematic appraisal of qualitative studies is particularly critical, as it directly shapes the evidence base used to inform health care practice, policy, and patient care [11]. Qualitative research in health not only helps to understand patient experiences, barriers, and preferences but also guides the development of interventions and health technologies that are responsive to real-world needs. Therefore, the rigor and consistency of quality assessment tools are deemed trustworthy and ultimately influence clinical decision-making, health policy recommendations, and the quality of care delivered to diverse patient populations.

### The Role of Artificial Intelligence in Research Quality Assessment

Artificial intelligence (AI) has received attention in the qualitative research field, offering new possibilities for many steps of the research process [12,13]. AI’s role in systematic reviews has been theorized and tested in the quantitative space, but its potential in the qualitative field is given less attention [14,15]. AI’s ability to work efficiently and at scale holds the potential for systematic appraisal of study quality, and the system’s consistency could reduce human bias and variability [16]. Assessments done by AI could also enable the inclusion or exclusion of pattern recognition, as an AI can be instructed to complete a task independently or dependent on previous tasks. However, while AI can process complex data, it may struggle with the nuanced, context-dependent nature of qualitative research.

Large language models are among the most popular AI tools for researchers today [17]. These advanced AI systems offer capabilities that could potentially revolutionize the process of qualitative research quality assessment, as they are pretrained to analyze vast amounts of textual data to enable nuanced analysis [18]. Furthermore, these systems are multilingual, potentiating the inclusion of a paper written in a language unknown to the human researcher. Finally, these specific systems can be fine-tuned or prompted (post training) to follow specific assessment criteria, suitable for the systematic quality assessment of qualitative studies [13]. The reliability and validity testing of the tools are problematic, as new versions or updates of the tools push their capabilities faster than the scientific community can assess their usefulness. The need to test these tools still exists, as the prevalence of its use is growing [19].

### Aims of the Study

Given the complex landscape of qualitative research quality assessment and the emerging potential of augmenting AI in research processes, this study aims (1) to evaluate and compare the performance of different AI models in assessing the quality of qualitative research studies using various assessment tools; (2) to compare the ratings given by 5 AI models (GPT-3.5, Claude 3.5, Sonar Huge, GPT-4, and Claude 3 Opus) when assessing qualitative studies; (3) to evaluate the interrater agreement among these AI models using 3 different assessment tools: Critical Appraisal Skills Programme (CASP), Joanna Briggs Institute (JBI), and Evaluative Tool for Qualitative Studies (ETQS); (4) to analyze how the exclusion of individual AI models affects the overall interrater agreement for each assessment tool; and (5) to identify specific items or criteria within these assessment tools that lead to prominent disagreements among the AI raters.

## Methods

### Overview

These models are chosen based on their diverse architectures and capabilities, which are crucial for a comprehensive analysis of AI augmentation in qualitative research. The selected models are included in Table 1.

**Table 1.** Overview of artificial intelligence (AI) models used for qualitative research appraisal.

Model	Developer	Release date	Size (B parameters)
GPT-3.5	OpenAI	2022	175
Claude 3.5	Anthropic	2024	Not disclosed
Sonar Huge	Perplexity AI, based on Llama 3.1	2024	405
GPT-4	OpenAI	2023	Not disclosed
Claude 3 Opus	Anthropic	2024	Not disclosed

This diverse selection of models detailed in Table 1 aims to identify which AI performance metrics are most beneficial for qualitative research quality assessment and to highlight areas where AI may complement or challenge each other. To evaluate the performance of these models, specific criteria will be used, including accuracy in coding, contextual understanding, and bias detection. The evaluation will use a standardized dataset of qualitative research papers to ensure a robust comparison across models.

### Quality Assessment Tools for Qualitative Research

#### Overview

The AI models will be instructed to use 3 widely recognized quality assessment tools for qualitative research. These tools have been comparatively analyzed in previous studies [20]. The CASP checklist was chosen for its widespread use and accessibility in various research fields and has been previously used in qualitative assessments and syntheses [21-23]. The JBI Critical Appraisal Checklist for Qualitative Research was chosen for its focus on the alignment between research objectives and methodological choices [22,24,25]. Finally, the ETQS was selected for its comprehensive approach to evaluating qualitative research integrity, offering a more nuanced assessment of methodological rigor. Each tool offers perspectives on qualitative research quality [20], which will facilitate a multifaceted assessment of the AI models’ ability to understand and evaluate different aspects of qualitative studies. The combination of these tools will provide a robust framework for comparing AI performance across various dimensions of qualitative research quality.

#### Assessment Process

Three peer-reviewed qualitative research papers have been selected as the source material for this study. These papers represent diverse topics within health and physical activity research, providing a robust basis for evaluating the AI models’ performance across varied contexts (Table 2).

**Table 2.** Summary of qualitative health studies evaluated by artificial intelligence models.

Study title	Year	Focus area	Methodology
Paper A: A qualitative study examining the validity and comprehensibility of physical activity items: developed and tested in children with juvenile idiopathic arthritis [26]	2019	Physical activity in children with juvenile idiopathic arthritis	Qualitative interviews
Paper B: “If only balls could talk...”: barriers and opportunities to participation for students with blindness and visual impairment in specialized PE [27]	2023	Participation barriers for students with visual impairments in PE^a^	Focus groups
Paper C: A qualitative study of exercise and physical activity in adolescents with pediatric-onset multiple sclerosis [28]	2019	Exercise and physical activity in adolescents with MS^b^	Semistructured interviews

a PE: physical education.

b MS: multiple sclerosis.

#### Application of AI Models to Each Assessment Tool

Each AI model will be tasked with applying all 3 quality assessment tools (CASP, ETQS, and JBI) to the selected studies. All papers are free text, and the AI models will be provided with the full text of each study as well as the assessment criteria for each tool. All AI-generated assessments will be collected and stored for analysis. AI-generated assessments will be formalized in a standardized format.

#### Repeated Assessments for Consistency

The repeated assessments for consistency are as follows:

1. Input preparation: The full text of each study will be provided to the AI models along with the complete assessment criteria for each tool. To ensure consistency, the input format will be standardized across all models.
2. Assessment protocol: AI models will be instructed to conduct a comprehensive quality assessment of each study using all 3 tools independently. Clear instructions will be provided to ensure the models understand the task requirements.
3. Structured output: To facilitate comparative analysis, AI models will be required to provide their assessments in a standardized format for each tool. This may include numerical scores, categorical ratings, and textual explanations.
4. Reasoning transparency: The AI models will be prompted to explain their reasoning for each assessment criterion, providing insights into their decision-making process and allowing for evaluation of their understanding of qualitative research principles.
5. Consistency evaluation: Each AI model will perform the assessment task multiple times to evaluate the consistency of their outputs and identify any variability in their assessments.
6. Data collection and storage: All AI-generated assessments, including explanations and any variations in repeated assessments, will be systematically collected and stored in a secure database for subsequent analysis. This will ensure data integrity and facilitate comprehensive evaluation.
7. Bias mitigation: To minimize potential biases, the order of presenting studies and assessment tools to the AI models will be randomized for each evaluation session.

### Ethical Considerations

All analyzed studies were previously published and had undergone their own ethical review processes. No new data were collected from individuals. The role of AI in the research process was disclosed, and all AI-assisted assessments were documented and stored securely. No personal or sensitive data were collected or processed. The research did not involve any intervention or interaction with human participants. The study posed no risk to individuals or groups, as it relied solely on secondary analysis of published material.

## Results

All AI models showed high “Yes” rates (75.9%‐85.4%), with Claude 3.5 achieving the highest affirmation (164/192, 85.4%), as detailed in Table 3. GPT-4 diverged significantly, showing lower agreement (“Yes”: 115/192, 59.9%) and elevated uncertainty (“Cannot tell”: 69/192, 35.9%). GPT-3.5 and Claude 3.5 exhibited near-perfect alignment (Cramer *V*=0.891; *P*<.001). Sonar Huge (“Yes”: 148/188, 78.7%) and Claude 3 Opus (145/191, 75.9%) demonstrated moderate consistency. GPT-4’s exclusion boosted CASP agreement by 20% (α=0.784 vs 0.653 baseline), highlighting its role as a variability driver. Statistical associations weakened with open-source models (Cramer *V*=0.496‐0.545), suggesting that architectural differences influence assessment patterns.

**Table 3.** Frequency of ratings by rater.

Model	Yes, n/N (%)	Cannot tell, n/N (%)	No, n/N (%)
GPT-3.5	158/192 (82.3)	34/192 (17.7)	0/192 (0)
Claude 3.5	164/192 (85.4)	28/192 (14.6)	0/192 (0)
Sonar Huge	148/188 (78.7)	33/188 (17.6)	7/188 (3.7)
GPT-4	115/192 (59.9)	69/192 (35.9)	8/192 (4.2)
Claude 3 Opus	145/191 (75.9)	38/191 (19.9)	8/191 (4.2)

Table 4 demonstrates GPT-3.5’s significant agreement across all models (*χ*²=47.0‐152.3; *P*<.001), with effect sizes revealing distinct patterns such as perfect concordance with Claude 3.5 (Cramer *V*=0.891; *χ*²_1_=152.3), moderate agreement with Sonar Huge (*V*=0.539; *χ*²_2_=54.6) and Claude 3 Opus (*V*=0.496; *χ*²_2_=47.0), and finally, a weaker association with GPT-4 (*V*=0.545; *χ*²_2_=57.0) despite shared commercial development.

**Table 4.** GPT-3.5 associations.

	Chi-square^a^ (*df*)	*P* value	Cramer *V*
Versus Claude 3.5	152.3 (1)	<.001	0.891
Versus Sonar Huge	54.6 (2)	<.001	0.539
Versus GPT-4	57.0 (2)	<.001	0.545
Versus Claude 3 Opus	47.0 (2)	<.001	0.496

a Chi-square test results showing associations between GPT-3.5 assessments and those of other artificial intelligence models.

The sensitivity analysis (summarized in Table 5) revealed tool-specific impacts of model exclusion on interrater agreement. For the CASP tool, excluding GPT-4 increased agreement by 20% (α=0.784), while Sonar Huge exclusion raised it by 18% (α=0.773), suggesting that these models introduce divergent interpretations of methodological rigor criteria. Conversely, JBI agreement improved most when excluding Sonar Huge (+18%; α=0.561) but dropped sharply without GPT-3.5 (−17%; α=0.398), indicating its stabilizing role for JBI appraisals. ETQS maintained the lowest baseline agreement (α=.376), with marginal improvements when excluding GPT-4 or Claude 3 Opus (+9%; α=0.409). This aligns with findings from Table 6, where ETQS criteria like policy implications (item 35) showed full-spectrum disagreements across models. Notably, proprietary models (GPT-3.5 or Claude 3.5) consistently supported consensus-building, as their exclusion reduced CASP or JBI agreement by 12%‐17%. This pattern mirrors architectural similarities observed in GPT-3.5 and Claude 3.5’s coding behaviors.

**Table 5.** Sensitivity analysis of interrater agreement (Krippendorff α)^a^ across model exclusion scenarios.

Model exclusion scenario	JBI^b^ (Δ%^c^)	CASP^d^ (Δ%)	ETQS^e^ (Δ%)
All 5 models	0.477	0.653	0.376
Exclude GPT-3.5	0.398 (−17)	0.572 (−12)	0.346 (−8)
Exclude Claude 3.5	0.468 (−2)	0.572 (−12)	0.356 (−5)
Exclude Sonar Huge	*0.561 (+18)* ^f^	*0.773 (+18)*	0.359 (−5)
Exclude GPT-4	0.494 (+3)	*0.784 (+20)*	0.409 (+9)
Exclude Claude 3 Opus	0.468 (−2)	0.572 (−12)	0.409 (+9)

a α values represent Krippendorff interrater reliability coefficient.

b JBI: Joanna Briggs Institute.

c Δ%=percentage change from full model agreement.

d CASP: Critical Appraisal Skills Programme.

e ETQS: Evaluative Tools for Qualitative Studies.

f Values in italics format highlight agreement improvements ≥10% across all tools.

**Table 6.** High-discrepancy Evaluative Tools for Qualitative Studies (ETQS) criteria across artificial intelligence models.

ETQS item	Criteria description	GPT-3.5	Claude 3.5	Sonar Huge	GPT-4	Claude 3 Opus	Disagreement score^a^
35	Generalizability to settings	Yes	Yes	Cannot tell	No	No	3
36	Generalizability to populations	Yes	Yes	Cannot tell	No	No	3
38	Policy implications	Cannot tell	Cannot tell	Yes	Cannot tell	No	2
43	Reviewer identification	Cannot tell	Cannot tell	Cannot tell	No	Yes	2
44	Review date verification	Cannot tell	Cannot tell	Cannot tell	No	Yes	2
8	Methodological framework alignment	Yes	Yes	No	Yes	Yes	2

a Number of distinct response categories (yes or cannot tell or no) per criterion.

**Figure 1.**
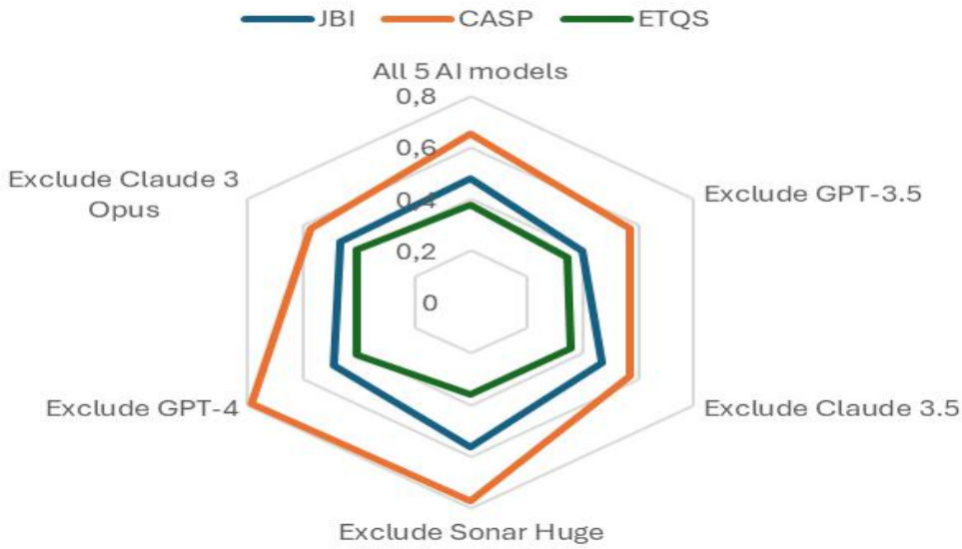
Radar-chart visualization upon model exclusion. AI: artificial intelligence; CASP: Critical Appraisal Skills Programme; ETQS: Evaluative Tools for Qualitative Studies; JBI: Joanna Briggs Institute.

Figure 1 illustrates how excluding specific AI models affects interrater agreement across 3 qualitative research assessment tools: JBI, CASP, and ETQS. The CASP tool demonstrated the highest baseline agreement (α=0.653), with notable improvements observed when GPT-4 or Sonar Huge was excluded, increasing agreement to 0.784 and 0.773, respectively. These findings suggest that GPT-4 and Sonar Huge may introduce variability in CASP assessments. In contrast, the exclusion of GPT-3.5, Claude 3.5, or Claude 3 Opus reduced agreement to 0.572, highlighting their role in fostering consensus.

For the JBI tool, excluding Sonar Huge resulted in the largest improvement in agreement (α=0.561), while removing GPT-3.5 led to a significant drop to 0.398, indicating that GPT-3.5 is a key contributor to maintaining consistency in JBI assessments. The ETQS tool exhibited the lowest baseline agreement (α=0.376), with marginal gains observed when GPT-4 or Claude 3 Opus were excluded, both increasing agreement to 0.409. This suggests that ETQS assessments are generally consistent across models, with GPT-4 and Claude 3 Opus introducing slight variability.

These results underscore the importance of model selection in AI-assisted qualitative research assessment, as certain models contribute more significantly to consensus, while others may introduce variability depending on the assessment tool used.

Table 6 highlights the ETQS criteria where AI models demonstrated the most significant disagreements in their assessments. Items such as generalizability to settings and populations (items 35 and 36) exhibited the full spectrum of possible responses (“Yes,” “Cannot tell,” and “No”), indicating substantial variability in model interpretation. Other items, including policy implications (item 38) and methodological framework alignment (item 8), also showed notable disagreement, albeit with fewer distinct response categories. These findings underscore the challenges AI models face in achieving consensus on nuanced qualitative criteria, particularly those requiring contextual or interpretive judgment.

## Discussion

### Principal Findings

This study reveals critical insights about AI’s role in qualitative research appraisal, particularly in health science contexts where methodological rigor directly impacts evidence-based practice [29,30]. All AI models demonstrated systematic affirmation bias, with “Yes” rates ranging from 75.9% to 85.4%, suggesting an inherent tendency toward favorable assessments regardless of the assessment tool (Table 3). Model-specific variability emerged as a key factor, particularly with GPT-4 diverging significantly (“Yes”: 115/192, 59.9%) compared to proprietary models like GPT-3.5 and Claude 3.5, which showed near-perfect alignment (Cramer *V*=0.891; *P*<.001) as detailed in Table 4.

Tool-dependent disagreements were evident, particularly with ETQS criteria like policy implications (item 35) and generalizability (item 36), which elicited the full spectrum of responses across models. This highlights current limitations of AI in contextual interpretation. In health research, such biases could distort evidence syntheses informing clinical guidelines or public health policies, especially for studies like Paper C (multiple sclerosis), where AI’s inability to contextualize structural barriers (eg, health care access disparities) risks undermining person-centered care models [31].

### Comparison to Prior Work

The findings of this study are consistent with emerging research on AI-augmented qualitative analysis. The consensus-building role of proprietary models mirrors previous findings regarding ChatGPT’s utility in thematic analysis [18]. AI’s challenges with nuanced criteria such as policy implications corroborate [13] known limitations in interpretive tasks critical for health policy design, such as balancing clinical efficacy with ethical or logistical constraints (eg, insurance coverage gaps in Paper A). The ongoing need for human validation supports the framework proposed by Hitch [12], which positions AI as a “team member” rather than a standalone evaluator. This approach is reinforced by the importance of patient-centered transparency in health care AI [32], where oversight mechanisms and impact on care experience directly influence trust.

### Strengths and Limitations

The strengths of the study are as follows:

- Standardized protocols: The use of standardized protocols and independent verification of AI outputs helped mitigate potential bias, especially given the lead author’s (AL) dual role as investigator and participant in Paper A.
- Diverse model selection: The inclusion of multiple AI models with varied architectures and capabilities facilitated a comprehensive analysis of AI’s potential and limitations in qualitative research appraisal.
- Tool variety: The application of 3 widely recognized assessment tools (CASP, JBI, and ETQS) provided a robust framework for evaluating AI performance across different dimensions of qualitative research quality.

The limitations of the study are as follows:

- Proprietary model opacity: The proprietary nature of commercial models (GPT-3.5 and Claude 3.5) obscures the architectural factors driving their consensus patterns, potentially masking biases that disproportionately affect vulnerable populations (eg, Paper B’s findings on physical education participation barriers).
- Dataset scope: The focused dataset of 3 health science papers limits generalizability, although the inclusion of pediatric and chronic disease contexts underscores current challenges for large language models in appraising life span–specific health narratives (Table 6).
- Author dual role: The lead author’s (AL) involvement as both investigator and participant in Paper A introduced potential interpretation bias, mitigated but not eliminated by standardized protocols.
- Absence of human expert ratings: The lack of human expert ratings prevents definitive conclusions about whether AI’s “favorable bias” reflects accuracy or systemic overestimation.

### Conclusions

This study demonstrates that AI models exhibit both promise and limitations as evaluators of qualitative research quality. This comprehensive analysis revealed 3 critical insights: first, affirmation bias was evident, with “Yes” ratings ranging from 75.9% to 85.4% across models, highlighting AI’s tendency to favor positive assessments, a pattern that could overstate the feasibility of interventions in health research. Second, model-specific variability emerged, as seen in GPT-4’s divergent ratings, which lowered CASP agreement by 20% and underscored the influence of model architecture on appraisal consistency. Third, disagreements were often tool-dependent, particularly for ETQS criteria like policy implications and generalizability, exposing current limitations in AI’s contextual interpretation.

The findings emphasize that AI cannot yet replace human judgment in nuanced qualitative appraisal but could enhance efficiency when strategically implemented. In health research, strong alignment of proprietary models (Cramer *V*=0.891) may expedite systematic reviews of patient experience studies, but their affirmation bias risks inflating confidence in underpowered qualitative evidence used for clinical guidelines. Open-source variability, while requiring oversight, could help counterbalance systemic optimism in AI-driven health syntheses.

Key limitations, including proprietary model opacity, which obscures biases affecting marginalized health populations, dataset scope constraints, and the author’s dual role in Paper A warrant cautious interpretation. The absence of human expert ratings is particularly consequential for health research, where patient narratives and clinician insights require a nuanced ethical appraisal that AI’s binary frameworks may oversimplify.

Future research should prioritize three areas: (1) health-specific AI training protocols emphasizing qualitative epistemology to better capture patient-centered care priorities, (2) benchmarking against expert panels to validate accuracy thresholds, and (3) establishing ethical frameworks for disclosing AI’s role in health evidence synthesis, ensuring transparency in policy recommendations. As qualitative methodologies evolve alongside AI capabilities, the path forward lies not in human-machine competition but in hybrid workflows that leverage AI’s scalability while preserving human expertise for contextual and interpretive tasks.

## Abbreviations

AI: artificial intelligence
CASP: Critical Appraisal Skills Programme
ETQS: Evaluative Tools for Qualitative Studies
JBI: Joanna Briggs Institute
